# Sickle Cell Disease: Current Drug Treatments and Functional Foods with Therapeutic Potential

**DOI:** 10.3390/cimb46060349

**Published:** 2024-06-12

**Authors:** Elisângela Gonçalves, Slim Smaoui, Miguel Brito, J. M. Oliveira, Ana Paula Arez, Loleny Tavares

**Affiliations:** 1 Global Health and Tropical Medicine (GHTM), Associate Laboratory in Translation and Innovation Towards Global Health (LA-REAL), Institute of Hygiene and Tropical Medicine, (IHMT), NOVA University of Lisbon (UNL) 1349-008 Lisbon, Portugal; a21001585@ihmt.unl.pt (E.G.); aparez@ihmt.unl.pt (A.P.A.); 2 Laboratory of Microbial and Enzymes Biotechnology and Biomolecules (LBMEB), Centre of Biotechnology of Sfax (CBS), University of Sfax-Tunisia, Road of Sidi Mansour Km 6, P.O. Box 1177, Sfax 3018, Tunisia; slim.smaoui@cbs.rnrt.tn; 3 Health Research Centre of Angola (CISA), Caxito, Angola; miguel.brito@estesl.ipl.pt; 4 H&TRC—Health & Technology Research Center, Escola Superior de Tecnologia da Saúde, Instituto Politécnico de Lisboa, 1990-092 Lisbon, Portugal; 5 School of Design, Management and Production Technologies Northern Aveiro, University of Aveiro, Estrada do Cercal, 449, 3810-193 Oliveira de Azeméis, Portugal; martinho@ua.pt; 6 EMaRT Group—Emerging Materials, Research, Technology, University of Aveiro, 3810-193 Aveiro, Portugal; 7 CICECO Aveiro—Institute of Materials, University of Aveiro, Campus Universitário de Santiago, 3810-193 Aveiro, Portugal

**Keywords:** sickle cell anemia, functional food, randomized, clinical trials, bioactive compounds

## Abstract

Sickle cell anemia (SCA), the most common form of sickle cell disease (SCD), is a genetic blood disorder. Red blood cells break down prematurely, causing anemia and often blocking blood vessels, leading to chronic pain, organ damage, and increased infection risk. SCD arises from a single-nucleotide mutation in the β-globin gene, substituting glutamic acid with valine in the β-globin chain. This review examines treatments evaluated through randomized controlled trials for managing SCD, analyzes the potential of functional foods (dietary components with health benefits) as a complementary strategy, and explores the use of bioactive compounds as functional food ingredients. While randomized trials show promise for certain drugs, functional foods enriched with bioactive compounds also hold therapeutic potential. Further research is needed to confirm clinical efficacy, optimal dosages, and specific effects of these compounds on SCD, potentially offering a cost-effective and accessible approach to managing the disease.

## 1. Introduction

**Figure 1:**
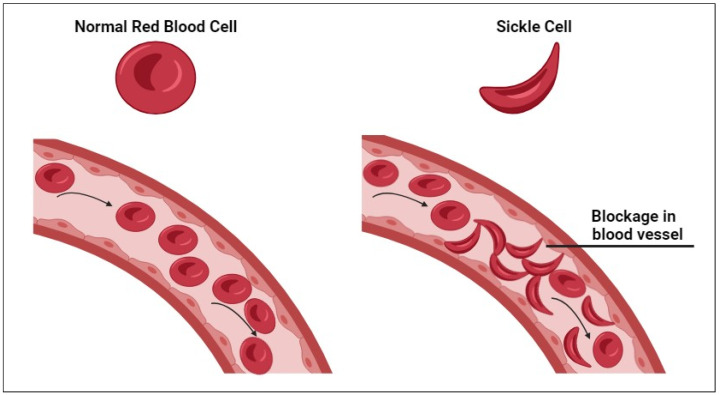
Comparison of normal and sickle red blood cells; sickle-shaped red blood cells obstruct blood flow in narrow blood vessels. Drawings were made by the authors using BioRender. Adapted from [4].

Sickle cell anemia (SCA), a severe genetic disorder that originated in Africa and spread globally through migration, is one of the world’s most common inherited blood diseases [1,2]. The World Health Organization (WHO) estimates that over 300,000 births occur with this condition every year worldwide [3]. It results from a mutation on human chromosome 11, in which adenine is replaced by thymine [1]. This change involves a single DNA base-pair substitution (GAG to GTG) in the beta-globin gene. This mutation alters the amino acid sequence of the beta-globin protein, replacing the hydrophilic glutamic acid at position six with the hydrophobic valine, resulting in hemoglobin S (HbS) with morphological abnormalities in the red blood cells in low oxygen pressure (Figure 1) [4].

The change in amino acid properties (hydrophilic to hydrophobic) within the β-globin chain contributes to the reduced solubility of HbS [5]. Therefore, the abnormal hemoglobin exhibits insolubility and polymerization in response to factors such as decreased oxygen tension, physical trauma, dehydration, stress, acidosis, or exposure to cold temperatures. Polymerized hemoglobin leads to the formation of rigid, inflexible, and easily damaged erythrocytes. These facts could lead to a reduction in lifespan, contributing to the development of a range of acute and chronic complications [6]. Studies examining the haplotypes associated with the sickle cell gene in Africa have revealed compelling evidence suggesting three distinct regions of origin for the mutation within the continent: the Central African Republic, Senegal, and Benin [1,7,8]. Individuals with SCD, homozygous for the βS mutation, typically exhibit the characteristic symptoms and complications associated with the condition. In very early infancy, SCD often presents as asymptomatic. Symptoms typically emerge with the decline in fetal hemoglobin, around 6 to 8 months of age [9]. However, as individuals age, various factors may intervene, influencing the symptomatic expression of SCD [10]. Individuals with SCA experience reduced red blood cell flexibility due to polymerization, resulting in rheological and biochemical alterations that impede blood flow, ultimately leading to vaso-occlusive crises (VOCs) [10,11]. Studies in genetic mapping and genome-wide association have identified specific genetic loci associated with SCA, including B-cell lymphoma/leukemia 11A (BCL11A), an Xmn1 variant located upstream of the hemoglobin subunit gamma 1 (HBG1), and the HBS1L-MYB intergenic region. These loci have been found to contribute to 20–50% of the variability observed in fetal hemoglobin (HbF) levels among individuals with SCA [12].

**Figure 2:**
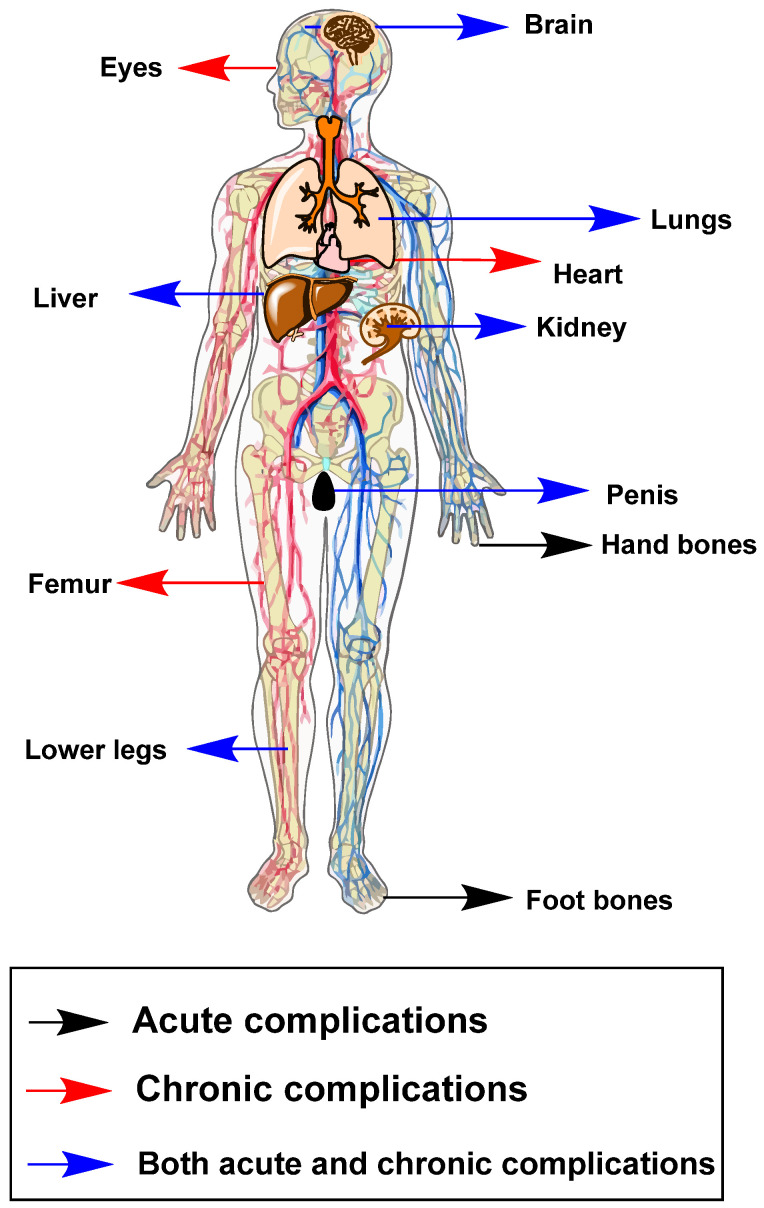
Acute and chronic complications of sickle cell disease in various body systems. Drawings were made by the authors (ChemDraw software, version 20.0.0.41). Adapted from [13].

SCD can manifest in various clinical features, including abnormal eye growth in the retina (proliferative retinopathy), tissue death due to blocked blood flow (vascular necrosis), prolonged painful erection (priapism), dilute urine (hyposthenuria), episodes of severely reduced red blood cell production (aplastic crises), kidney problems (nephropathy), and lung disease [5]. For individuals with SCD, episodes of severe pain caused by blocked blood vessels (vaso-occlusive crisis) are the most frequent reason for emergency department visits and hospital admissions [9]. Other symptoms manifested in sickle cell anemia carriers include severe bacterial infections, necrosis (tissue death), acute chest syndrome, ischemic and hemorrhagic stroke, sepsis, hemolytic crises, and functional asplenia [1]. SCD affects various body parts, causing a range of symptoms and complications, which can be acute, chronic, or a combination of both (Figure 2).

Many techniques and assays are used to identify and track SCD. These consist of hemoglobin electrophoresis, isoelectric focusing, peripheral blood smears, complete blood cell counts, hemoglobin solubility tests, and high-performance liquid chromatography. Comprehensive information regarding genetic mutations can be obtained using genetic testing options such as restriction fragment length polymorphism, PCR-based methods, and DNA sequencing. Flow cytometry, mechanical sickle cell differentiation, lateral flow immunoassay, and density-based separation are examples of emerging approaches. Further diagnostic capabilities are provided by additional techniques such as pyrosequencing, the hemoglobin solubility test on paper, and sensor-based technologies including electrical impedance sensors and optofluidic resonators based on fluorescence [14]. The current approach to SCD management prioritizes symptom relief and complication prevention in the absence of a definitive cure, with efforts to develop effective treatments facing significant challenges and slow progress [15,16]. In this sense, different drugs targeting diverse biochemical pathways have been developed for SCD [17]. Despite promising leads in some cases, many fail to demonstrate significant benefits in clinical trials [5]. Even for those showing potential, limited availability creates a substantial access barrier due to its high costs, particularly in low-income countries [5]. In Africa, where traditional healing practices are prevalent, many people with SCD rely on plant-based remedies (phytomedicines) as a primary source of healthcare, especially for painful episodes and other SCD complications [5,18]. Studies have shown that many medicinal plant species from both developing and developed countries possess promising antisickling properties, suggesting their potential for treating and managing sickle cell anemia [19]. Yembeau et al. [19] conducted an ethnobotanical exploration focusing on plants commonly utilized by the populations of Nigeria’s southwestern region for disease management. The study identified 44 plant species with promising properties for the development of new medications to manage various diseases [20]. Plants contain abundant bioactive compounds that hold promise as antisickling agents capable of neutralizing free radicals, which could potentially reduce oxidative stress on red blood cells, leading to a decrease in hemolysis (red blood cell destruction) and a longer lifespan for these cells [5]. Therefore, incorporating foods rich in bioactive compounds into the diets of people with SCD might offer a complementary approach to manage the condition. These functional foods could potentially improve clinical health by reducing the risk or severity of complications associated with the disease [5].

This pilot study presents a comprehensive review analyzing randomized, double-blind placebo-controlled trials (RCTs) across Phase I, II, and III for interventions in SCD, focusing on various treatment modalities. Randomization aims to eliminate both unconscious and deliberate human influence on the assignment of subjects to different groups. Blind assessment ensures that treatment and analysis of outcomes are not colored by prejudice [21]. This work also summarizes and discusses the findings from these trials while also exploring the potential of functional foods and herbs rich in bioactive compounds as therapeutic agents for SCD management.

## 2. Methods

**Figure 3:**
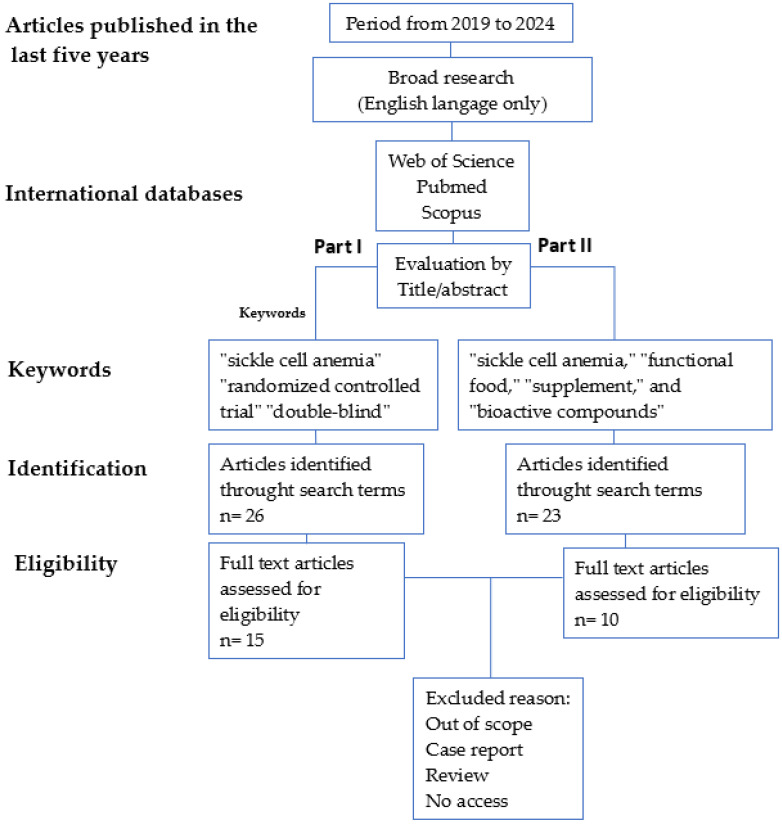
Flow diagram of systematic review selection criteria.

This review aimed to identify relevant research on treatments for sickle cell anemia. A comprehensive search strategy was employed across international databases like Scopus, PubMed, and Google Scholar, encompassing the period from 2019 to 2024. The search was divided into two distinct parts (Figure 3). Part I focused on identifying double-blind randomized controlled trials investigating potential treatment compounds. Keywords used in this search included “sickle cell anemia,” “randomized controlled trial”, and “double-blind,” to ensure robust study design. Additionally, articles were included if they employed an experimental design, had relevance to both food and pharmaceutical applications, and were published in English. This rigorous selection process yielded 15 articles that met the established criteria. Part II of the search strategy explored the use of supplements as functional food additives for patients with sickle cell disease. The keyword search employed terms like “sickle cell anemia”, “functional food”, “supplement”, and “bioactive compounds” to identify relevant studies. The inclusion criteria mirrored those of Part I, ensuring the identification of articles with experimental designs, applicability to both food and pharmaceutical domains, and publication in English. This search identified 10 articles that met the established criteria. The impact of bioactive compounds in SCD management is also present in Section 5.

## 3. Review of Randomized, Double-Blind Clinical Trials for Sickle Cell Disease Treatments

The US Food and Drug Administration (FDA) recognizes four treatment options for preventing and alleviating complications associated with SCD: hydroxyurea (HU), voxelotor (Oxbryta/GBT440), L-glutamine, and crizanlizumab [5]. These medications represent significant advancements in managing SCD, but researchers are relentlessly pushing the boundaries to improve treatment effectiveness and patient outcomes. One key area of exploration involves investigating novel therapeutic compounds, potentially leading to a future where more patients with SCD can experience improved quality of life and a reduced burden of complications. Several promising candidates are currently being evaluated in rigorous clinical trials, including randomized, double-blind, placebo-controlled Phase I, II, and III studies (Table 1).

These trials are designed to ensure the objectivity and generalizability of the findings by eliminating subjectivity and unconscious influences.

The addition of HU, L-glutamine, crizanlizumab, and voxelotor to the treatment options for SCD represents a significant step forward [16]. HU, a drug that effectively increases fetal hemoglobin (HbF) production, has emerged as a pivotal option in managing SCA, demonstrating efficacy and practicality [37]. HbF, a type of hemoglobin present in fetal red blood cells, has a more flexible structure than adult hemoglobin, allowing it to bend and deform without sickling even under conditions of low oxygenation. This characteristic is crucial in the pathogenesis of SCD [38]. However, HU can have some side effects, and researchers continue to explore new treatment options. HU is a strong inhibitor of the enzyme ribonucleotide reductase within the cell and converts ribonucleotides into deoxyribonucleotides, which are essential for DNA synthesis and repair. It has been used for several decades in the treatment of various disorders, especially myeloproliferative neoplasms, chronic myeloid leukemia, and the human immunodeficiency virus (HIV) [39]. Over time, speculation arose that HU could potentially be carcinogenic and harmful to DNA. With this, the widespread use of HU was reduced, and many patients stopped receiving treatment with this drug. Research suggests that HU may not be equally effective for all patients with SCD. Additionally, safety concerns have been raised regarding potential harm to a developing fetus (embryofetal toxicity) and the suppression of bone marrow activity (myelosuppression) [5,40].

The guidelines published by the National Institutes of Health, the American Society of Hematology, and the British Society of Hematology all recommend HU as the standard treatment for SCA, but it is important to investigate its genotoxic and carcinogenic potential [41]. Abdullahi et al. [31] conducted a double-blind, randomized controlled trial assessing the efficacy of low-dose (10 mg/kg/day) versus moderate-dose (20 mg/kg/day HU in 220 children with SCA and abnormal transcranial Doppler velocities. The study revealed no significant difference in stroke incidence between the groups over a 20-month period. This finding suggests that a lower dose of HU might be similarly effective for stroke prevention in this pediatric population. The research findings potentially influenced clinical practice guidelines in three Nigerian states, impacting an estimated 40,000 children at risk of stroke.

Fish oil is a rich source of omega-3 fatty acids, specifically two major types: docosahexaenoic acid (DHA) and eicosapentaenoic acid (EPA). These essential fats fall under the broader category of polyunsaturated fatty acids and are vital for maintaining human health [22,42]. Giriraja et al. [22] elaborated a study where ten adults with SCD were given an omega-3-enriched food, daily, for a month. The authors concluded that the omega-3-enriched food reduce sickle cell-related inflammation. Abdelhalim, Murphy, Meabed, Elberry, Gamaleldin, Shaalan, and Hussein [23] conducted a randomized, double-blind clinical trial and investigated the effects of omega-3 fish oil supplementation in 165 patients. Participants received either 1000 mg of omega-3 fish oil (containing 400 mg EPA and 300 mg DHA) or a daily dose of 2800 IU vitamin D for 10 months. The study found a significant increase in both serum high-density lipoprotein (HDL) and low-density lipoprotein (LDL) cholesterol levels in the omega-3 group compared to the control group. Additionally, the omega-3 group experienced a notable decrease in the number and severity of pain crises. People with SCD commonly exhibit reduced antioxidant defenses in their bloodstream, potentially due to lipid peroxidation resulting from interactions with ferroptosis and compromised antioxidant capacity. Additionally, EPA and DHA act as powerful anti-inflammatory agents that can modulate pain [42].

The human body relies on a multitude of vitamins, and vitamin A stands out for its versatility. It influences a remarkable range of processes throughout life, from enabling healthy development before and after birth to supporting vision, reproduction, and tissue repair. Additionally, vitamin A strengthens our immune system, acting as a key player in overall health [24,43]. In American children with SCD, inadequate levels of vitamin A are related with increased rates of hospitalization, lower development, and inferior hematologic status (hemoglobin 7.9 vs. 8.5 g/dL) compared to children with appropriate vitamin A levels [44]. Brownell et al. [24] conducted an 8-week study in which twenty healthy infants with hemoglobin-SS illness received either 3000 or 6000 IU/d of retinol orally. The authors reported minor improvements in exploratory nutritional, hematologic, and muscular results after supplementation. They recommend additional research in the form of a larger-sample longitudinal placebo-controlled study that considers the use of HU.

Folic acid, also known as vitamin B9, is commonly prescribed to SCD patients to support red blood cell production [45]. Muga et al. [25] conducted a randomized double-blind, active-controlled, clinical trial with 61 individuals for 4 weeks, where the intervention group received 30 capsules of folic acid 500 microgram and 60 capsules of EvenFlo 500 mg. The authors concluded that EvenFlo, a nutritional supplement, shows promise in managing SCD when combined with folic acid. The results suggest that EvenFlo may be beneficial for increasing hemoglobin levels, improving weight management in patients with SCD, and potentially reducing the frequency of crisis episodes.

Vitamin D, a fat-soluble substance essential for the human body, primarily regulates calcium and phosphorus levels to maintain bone health, and also plays important roles in the cardiovascular, immune, and pancreatic systems, as well as in muscle, brain function, and cell cycle control [46]. Grégoire-Pelchat et al. [26] investigated the effectiveness of vitamin D supplementation in raising serum levels of 25-hydroxyvitamin D (25(OH)D) in 38 children with SDC. Participants received either a high-dose vitamin D bolus combined with daily 1000 IU vitamin D3 or daily supplementation alone. Additionally, researchers assessed various health markers, including calcium levels, bone markers, musculoskeletal pain, quality of life, and hematological parameters. The study found that the combined approach was more effective in increasing 25(OH)D levels compared to daily supplementation alone.

Arginine is a nutritional amino acid and the obligate substrate for nitric oxide (NO) production [27,28]. Studies suggest that impaired L-arginine metabolism and NO availability contribute to the vascular dysfunction observed in SCD [28,47]. NO acts as a potent vasodilator, impacting various vascular and circulating blood cell functions such as platelet aggregation inhibition, adhesion molecule down-regulation, and ischemia-reperfusion injury modulation, all crucial pathways affected during VOCs [27,48,49]. Onalo et al. [27] investigated the effects of oral L-arginine supplementation in children with sickle cell disease (SCD) hospitalized for vaso-occlusive pain crises (VOC) in Nigeria. For 24 months, participants received either oral L-arginine hydrochloride (100 mg/kg three times a day) or a control treatment. This study provided the evidence that daily oral arginine supplementation may improve pain control and reduce the need for pain medication in children experiencing VOCs associated with SCD. Onalo et al. [28] investigated the effects of oral L-arginine supplementation in 47 children (aged 5–17 years) hospitalized with severe pain and/or acute chest syndrome. The study compared daily L-arginine (300 mg/kg/day in three divided doses) to a placebo over 22 months. Notably, treatment with L-arginine led to a greater reduction in key cardiopulmonary hemodynamic markers, including pulmonary artery pressure, compared to a placebo. These findings suggest that L-arginine supplementation may offer benefits for improving cardiovascular health in SCD patients experiencing vaso-occlusive episodes and acute chest syndrome.

Voxelotor is a recently approved drug often used as an alternative for patients unresponsive to HU, with its long-term efficacy and safety currently under investigation [50,51]. It was recently approved by the FDA for the treatment of SCD in children aged 12 and older [50,51]. This molecule binds to hemoglobin, boosting its oxygen-carrying capacity (affinity) for better delivery throughout the body. It also stabilizes hemoglobin, preventing the abnormal clumping (sickling) that causes complications. Its daily oral administration has been shown, both in vitro and in vivo, to reduce the sickling of red blood cells, improve blood viscosity, and improve red blood cell deformability [30]. Additionally, it prolongs the half-life of red blood cells, reducing anemia and hemolysis. The drug is generally well-tolerated, although it may cause moderate adverse effects such as headache, diarrhea, nausea, and arthralgia [50]. Hutchaleelaha et al. [29] evaluated the safety, tolerability, pharmacokinetics, and pharmacodynamics of voxelotor for the first time in humans. The study included both healthy volunteers and individuals with SCD. Twenty-four healthy volunteers received varying daily doses of voxelotor (300, 600, or 900 mg) or a placebo for 15 days. The findings revealed favorable tolerability profiles in both SCD patients and healthy volunteers. Moreover, the study provided evidence indicating that voxelotor enhances hemoglobin–oxygen affinity, a critical mechanism believed to offer therapeutic benefits in SCD. Furthermore, assessments of erythropoietin levels, exercise tolerance, and hematologic parameters consistently demonstrated normal oxygen delivery during both resting periods and physical exertion. Vichinsky et al. [30] also investigated the effects of voxelotor in SCD. The study involved 274 participants randomly assigned to receive either a daily dose of 1500 or 900 mg of voxelotor, or a placebo, for 24 weeks. The researchers found that voxelotor significantly increased hemoglobin levels and reduced the frequency of worsening anemia and hemolysis compared to the placebo group. Notably, despite the rise in hemoglobin, there was no observed increase in VOC rates, suggesting that voxelotor may improve hemoglobin levels without negatively impacting blood viscosity.

Some intravenous (IV) medications also play a role in managing pain associated with SCD [32]. Notably, the FDA has approved IV acetaminophen for children over two years old to manage mild to severe pain, both with and without opioids [52,53]. While IV diclofenac remains the current standard treatment for managing skeletal VOCs in SCD, its use requires careful monitoring due to potential side effects [32]. These include respiratory depression, severe constipation, and the risk of dependence with frequent use.

Opioid medications, although effective for pain relief, often have limited availability and require similar monitoring due to their side effects [32,52]. Panda, Mishra, Patra, Nayak, and Panda [32] compared the effectiveness of IV acetaminophen and diclofenac sodium in managing skeletal VOCs in 104 children with SCD. The study found that IV acetaminophen (10 mg/kg/dose) led to several improvements compared to IV diclofenac sodium (1 mg/kg/dose) administered over two months. These included a greater reduction in pain scores (50%), a decrease in the number of medication doses needed to achieve pain relief within 24 h, and a faster reduction in pain scores within the first hour. Based on these findings, the researchers concluded that IV acetaminophen may be a preferable alternative to IV diclofenac [37]. Other studies are currently in progress to explore therapeutic alternatives, aiming to identify new pharmacological agents of different natures and potential drug candidates for managing this disease. Meanwhile, a wide range of compounds are being investigated to complement treatment and promote significant improvements in the quality of life of individuals affected by this condition. One example of these advances is the monoclonal antibodies crizanlizumab (adakveo) and canakinumab [33,54]. Crizanlizumab, a humanized monoclonal antibody, has been approved for the treatment of SCD in patients aged 16 and older. Its mechanism of action includes attenuating VOCs by binding to P-selectin and blocking its interaction with other molecules, preventing the adhesion of red blood cells. Crizanlizumab received FDA approval in 2019 to reduce VOC frequency in patients with SCD in adults and children SCD patients 16 years of age and older [15,37]. This approval was based on positive results from the SUSTAIN trial, which demonstrated a 45.3% reduction in annual pain crises for patients receiving crizanlizumab compared to a placebo group [55]. Notably, the benefit was even greater for patients not taking HU, with a 50% reduction in annual crisis rate compared to the placebo. However, preliminary data from the phase III STAND clinical trial, released in early 2023, did not show a statistically significant difference in annual vaso-occlusive crisis rates between crizanlizumab and placebo groups. This lack of efficacy in the STAND trial led to the European Medicines Agency’s decision to remove crizanlizumab from their list of approved SCD medications [56].

*Canakinumab* is a human monoclonal antibody that acts by blocking interleukin-1 beta (IL-1β), a pro-inflammatory protein involved in various chronic inflammatory conditions. In addition to being beneficial in the treatment of various rheumatic diseases, a reduction in cardiovascular events has been observed after myocardial infarction. Regarding SCA, it is being investigated as a possible therapeutic option due to its potential to modulate the inflammatory response, which plays an important role in the pathogenesis of this disease [33].

Rees, Kilinc, Unal, Dampier, Pace, Kaya, Trompeter, Odame, Mahlangu, and Unal [33] investigated the effects of *canakinumab* in adolescents and young adults with SCA (HbSS or HbSβ0-thalassemia). The study involved 49 participants aged 8 to 20 years with a history of acute pain episodes. The participants received either 300 mg subcutaneous *canakinumab* or a placebo, administered six times over a set period. The study found *canakinumab* to be well-tolerated with no serious adverse events (SAEs) attributable to the treatment and no unexpected safety concerns. Notably, the authors observed that canakinumab’s selective blockade of IL-1β inflammation appeared to yield therapeutic benefits, particularly by reducing fatigue, a significant symptom in SCA patients.

Ticagrelor is an antiplatelet drug widely used as a P2Y12 receptor antagonist, essential for platelet activation [57]. It is employed to prevent cardiovascular events like heart attacks and strokes in adults with coronary or cerebrovascular artery disease [58]. Its action involves blocking the P2Y12 ADP receptor on platelets, inhibiting their activation and aggregation, thereby reducing blood clot formation, and helping to prevent adverse cardiovascular events [58]. Currently, clinical studies are underway to investigate the use of ticagrelor in SCD treatment, reducing and contributing to complications such as VOCs and strokes, which have shown satisfactory tolerance and safety [59,60]. Heeney, Abboud, Githanga, Inusa, Kanter, Michelson, Nduba, Musiime, Apte, and Inati [34] investigated ticagrelor’s effectiveness in reducing VOCs in children with SCD. Participants received ticagrelor or a placebo for 12 months, with varying doses based on weight. However, the trial was stopped early after 8 months due to safety concerns. The data monitoring committee determined that the potential risks of continuing outweighed any potential benefits of ticagrelor. This unfortunately adds ticagrelor to a growing list of unsuccessful medications for SCD, which includes senicapoc, prasugrel, regadenoson, sevuparin, poloxamer 188, olinciguat, and rivipansel [35].

Ketamine, a medication that blocks a specific receptor in the nervous system (NMDA receptor), shows promise in reducing a condition called opioid-induced hyperalgesia. This occurs by potentially preventing nerve cells in the spine from becoming overly sensitive to pain signals [35,61]. It operates on glutamate and NMDA receptors, influencing the peripheral pain sensitization process along pain pathways [61,62]. Additionally, ketamine is theorized to impact neural plasticity in both the NMDA receptors and spinal pathways, thereby impeding the transmission of stimuli generated towards the central nervous system [62]. Alshahrani, AlSulaibikh, ElTahan, AlFaraj, Asonto, AlMulhim, AlAbbad, Almaghraby, AlJumaan, AlJunaid, Darweesh, AlHawaj, Mahmoud, Alossaimi, Alotaibi, AlMutairi, AlSulaiman Pharm, Alfaraj, Alhawwas, Mbuagbaw, Lewis, Verhovsek, Crowther, Guyatt, and Alhazzani [35] investigated the effectiveness and safety of a single, low-dose ketamine infusion (at 0.3 mg/kg) for managing acute VOC in adults with SCD. The study involved 278 adults with SCD over a 12-month period. The findings suggest that ketamine provided significant pain relief, allowing patients to require lower cumulative morphine doses. Importantly, the study found no major safety concerns with ketamine use. The authors recommend further research to explore the effectiveness and safety of repeated ketamine dosing or continuous infusion for managing VOCs in adults with SCD. Additionally, they suggest investigating the potential benefits of combining ketamine with opioids compared to opioid use alone.

Another treatment under study is the use of isoquercetin, a flavonoid found in various sources such as citrus fruits, onions, apples, and teas [63]. It is the orally available form of quercetin and it possesses the glycosidic form [63]. It has been studied for its antioxidant, anti-inflammatory, antimicrobial, and antiviral properties, and its use has improved thrombosis biomarkers in cancer patients without inducing bleeding [36,64]. Therefore, some studies are testing its efficacy and safety in regulating thromboinflammatory processes in SCD [36,64]. *Sevuparin*, a drug derived from heparin that exhibits broad preclinical activity, targets relevant targets in VOCs, such as P- and L-selectin, thrombospondin, von Willebrand factor, and fibronectin [65]. Although the anticoagulant activity of this drug has been removed, its anti-adhesive properties have been preserved. This compound has been shown to be a potent anti-adhesive agent, both in in vitro and in vivo studies, which is crucial in the context of VOCs in SCD. It helps prevent or reduce blood vessel obstruction, a common phenomenon in this condition due to the abnormal adherence of sickle cells to each other and vessel walls [66,67]. Lizarralde-Iragorri, Parachalil Gopalan, Merriweather, Brooks, Hill, Lovins, Pierre-Charles, Cullinane, Dulau-Florea, Lee, Villasmil, Jeffries, and Shet [36] investigated the effectiveness of isoquercetin in reducing inflammation associated with blood clots (thromboinflammation) in SCD. The study involved 46 patients receiving either a placebo or 1000 mg of isoquercetin daily for 28 to 35 months. The researchers observed a significant reduction in several biomarkers linked to thromboinflammation in patients taking isoquercetin compared to the placebo group. These findings suggest that future trials are warranted to explore the potential benefits of higher isoquercetin doses and longer treatment durations for managing thromboinflammation in SCD patients.

## 4. Functional Food Exploited in SCD Management

Functional foods, often referred to as nutraceuticals, are foods that are rich in bioactive substances that improve the health of their consumers [68]. According to Granato, Barba, Bursać Kovačević, Lorenzo, Cruz, and Putnik [69], functional foods encompass both natural and processed options that, when incorporated regularly into a varied diet, may offer additional health benefits beyond basic nutrition. The following requirements are the primary conditions for an ingredient or product meeting a certain functional claim on a food label [69]: (i) food security; (ii) accessible without a prescription (or guidance from a doctor); and (iii) proof that consuming it on a regular basis in a balanced diet has health benefits. Luvián-Morales, Varela-Castillo, Flores-Cisneros, Cetina-Pérez, and Castro-Eguiluz [70] define a functional food as one that delivers a variety of health benefits, including enhancing overall physical well-being and reducing the risk of diseases.

The exploration of functional foods represents a dynamic and rapidly evolving field within nutritional science. This section explores the most current research and study results about bioactive substances present in nutrients and their possible health advantages in SCD. Referred to as antisickling factors, biological compounds from foods and comestible and medicinal plants could enhance the health-related quality of life in SCD. These matrixes are abundant in phenolics, vitamins, minerals, proteins, amino acids, and unsaturated fats, and possess antisickling activity [71,72]. Researchers are exploring the potential of antioxidant-rich compounds in managing SCD. These substances may operate by reducing the body’s oxidative stress levels, a factor believed to intensify the severity of the illness [73]. Their ability to lower oxidative stress makes them a promising treatment option for sickle cell complications [42,74].

Increasing antioxidant intake may help SCD patients by supporting cellular renewal and promoting the formation of red blood cells [75]. For example, significant components in vegetables, phenolics have promising impact on human health. As an illustration, the *Carica papaya* was employed as a substitute therapeutic agent for SCD [74]. Polar phenolic compounds viz. phenolic acids kaempferol and quercetin were detected and characterized in papaya leaves [76]. The extract has an antisickling impact and a substantial membrane stabilizing (protective) effect. Another study conducted by Famojuro, Adeyemi, Ajayi, Fasola, Fukushi, Omotade, and Moody [77] reported that two isolated compounds from the root of *Combretum racemosum* P. beauv (*Combretaceae*) showed effective SCD management. These compounds are proved to trigger red cells’ membrane-bound enzymes Na^+^, K^+^-ATPase, and Ca^2+^-ATPase, which are engaged in the sickling process. Ahajumobi and Asika [78] demonstrated that 2-hydroxymethylbenzoic acid has an antisickling impact and could stabilize the erythrocytes membrane. It has been revealed that some amino acids could avoid sickling by disturbing the erythrocyte membrane, provoking intensification in the cell volume of the erythrocyte, and consequently decreasing the intracellular hemoglobin level [79,80]. Among the described amino acids, phenylalanine has been revealed to be the most efficient [81]. L-arginine reduces oxidative stress and L-Glutamine diminishes inactive energy expenditure [82]. Niihara [83] reported a phase 3 trial demonstrating efficacy in reducing acute complications of SCD, leading the FDA to approve pharmaceutical-grade L-glutamine (Endari, Emmaus Medical) as a prescription medication for adults and children aged 5 and above.

Daak et al. [84] concluded that omega-3 fatty acids reduce thrombotic activities and the level of episode pain. In this sense, elevated protein and L-Arginine supplements and n-3 fatty acids to Heinz body (denatured Hb) have shown a noteworthy lowering in inflammation, oxidative stress, red cell density, and pain episodes, while enhancing microvascular function. In addition, numerous dietary supplements, like thiocyanate, possess a vast ability to prevent erythrocytic deformations in the management of sickle cell disease [76].

Vitamin E and β-carotene are obtainable in foods such as vegetable oils, nuts, seeds, breakfast cereal, and fortified fruit juices, margarine, and spreads. Some investigations have stated that high levels of vitamin E decrease oxidative stress-induced erythrocyte injury [85,86]. Physiologically, vitamin E lessens lipid peroxidation and amends erythrocyte membrane stability [87]. On the other hand, ascorbic acid is lengthily circulated in nature, typically abundant in leafy vegetables and fresh fruits. Vitamin C normalizes attenuated hemodynamic alterations brought on by postural corrections and inhibits the development of Heinz body (denatured Hb) in vitro in sickle red blood cells [76]. Similarly, vitamin B9 (folic acid) could manage HbS; this management is predicated on stopping the absence from the amplified folate turnover [88]. In this way, vitamin B9 supplementation could reduce the high risk of endothelial damage.

Regarding minerals, Mg adjunct could reduce the number of dense erythrocytes and improve the erythrocyte membrane transport abnormalities of individuals with SCD [5,89,90]. Research suggests that maintaining magnesium levels within the recommended range for patients, with regular monitoring, might be associated with a lower risk of mortality [91]. Zn can be found in plentiful foods, including seafood (as herring and oysters), beans and peas, and grains. Zn could enhance thymulin activity, reduce bacterial contamination occurrence, and cure painful disasters [92].

Fruits and vegetables contain a diverse range of functional compounds, including carotenoids, vitamin C, fiber, magnesium, and potassium. These components work together, sometimes in cooperation (synergistically) and sometimes in opposition (antagonistically), to contribute to overall health benefits. Strong evidence supports current dietary recommendations that encourage increased consumption of fruits and vegetables, aiming for at least five servings daily [76]. In addition, a wide range of constituents found in functional food, each with unique physiological effects, has been demonstrated by recent research. For example, black beans (*Phaseolus vulgaris* L.), *Fragaria vesca* L., bitter *Garcinia kola*, *Annona muricata* L., and *Azadirachta indica* J. have been shown to be able to manage SCD [76,93]. Some studies have concluded that vitamins C and E and minerals viz. Zn and Mg could reduce the percentage of irreversibly sickled cells [94,95].

## 5. Impact of Bioactive Compounds in Sickle Cell Disease Management

Researchers are exploring the potential of various natural plant compounds for SCD (Table 2). Before hydroxyurea was available in Africa, people with SCD depended on hospital-based supportive treatments and home-based herbal medicines, with several medicinal plants identified that reactivated γ-gene transcription through different cellular mechanisms [96]. Commonly used herbs possess antisickling properties and are primarily utilized to prevent VOCs [96]. The potent components found in medicinal plants and natural compounds, termed antisickling agents, abundant in aromatic amino acids, phenolic compounds, and antioxidants, serve as antioxidant therapy to alleviate the complexities of sickle cell anemia, exhibiting protective effects such as shielding against lipid peroxidation in red blood cells, enhancing glutathione levels (GSH), and diminishing levels of reactive oxygen species (ROS) [97]. Therefore, it was concluded that herbal medicines have substantial potential in the therapeutic management of sickle cell disease, with the possibility of addressing both the underlying causes and the symptoms of the condition [74].

One promising example is niprisan, a plant-based medicine with antisickling properties. Studies suggest it may inhibit the polymerization of HbS, a key factor in SCD complications [112,113].

Patent (US5800819A) describes the phytochemical composition of a product containing a mixture of four plant materials: *Piper guineense* seeds and *Pterocarpus osun*, *Eugenia caryophyllus*, and *Sorghum bicolor* extracts. The mixture has been used for the treatment of SCD [114]. The extracted fraction from Ciklavit (*Cajanus cajan*) extract, rich in essential amino acids, vitamin C, and Zn, may decrease the occurrence of painful crises and mitigate the negative impact of SCA on the liver [71]. The authors reported that the mechanism of action remains to be determined [115].

The rising consumer demand for functional foods, those offering health benefits beyond basic nutrition, has spurred a surge of innovation within the food industry [116,117]. Nevertheless, a significant challenge persists ensuring the consistent quality and effectiveness of these enriched products throughout their entire journey, from initial preparation and processing to transportation, storage, and, finally, consumption by the end user. Bioactive compounds are chemically unstable, prone to degradation, volatilization, and oxidation, leading to a loss of their biological activities when exposed to harsh environmental conditions like high temperatures and the presence of oxygen and light [116,117]. Hence, their vulnerability to degradation constrains their direct application in food products and limits their potential health benefits. Encapsulation technology stands out as a potentially transformative solution. By encapsulating bioactive compounds within a protective shell, this technology not only shields them but also enhances their essential properties, such as solubility, stability, and bioavailability, and their antioxidative effects. Moreover, encapsulation facilitates the controlled release of these beneficial compounds, thereby maximizing their positive impact on human health [116,117]. The field of natural products for sickle cell disease (SCD) management holds immense promise. Advancements in nutrigenomics, digital health, omics technologies, personalized nutrition, and sustainability offer exciting avenues for future research and development. Exploring the nutritional properties of functional foods and harnessing their therapeutic potential can establish a path toward a healthier, more resilient future. Including health-related statements on functional food product labels is a key method of educating consumers about the relationship between nutrition and health. However, regulations are essential to oversee these claims, as they significantly influence consumer food choices.

## 6. Conclusions

Current treatments for SCD have limitations. Therefore, well-designed clinical trials are crucial to validate the effectiveness of new therapies before widespread use. Recent trials with pharmaceutical interventions for both acute and chronic symptoms of SCD show promise. Despite the encouraging findings from these trials, there remains a necessity for further investigations. Functional foods enriched with bioactive compounds hold particular interest for SCD management, especially in resource-limited regions where traditional herbal remedies are prevalent. These foods may offer therapeutic, antisickling, antioxidant, and inhibitory effects, acting as potential combatants against sickle cell disease crises. This highlights the urgent need for clinical studies to assess the potential benefits of such nutrition-based approaches, focusing on commonly used medicinal plants to maximize their feasibility and biological impact. Research into the role of food in managing SCD demands further research. Plants with a long history of medicinal use present a promising and practical avenue for exploration. Rigorous evaluation of functional foods enriched with beneficial compounds could lead to the discovery of new and accessible treatment options for individuals living with SCD, particularly in regions with limited resources.

## Figures and Tables

**Table 1:** Randomized double-blind controlled studies of different products used in the treatment of sickle cell disease.

Product	Objective	Formulation and Dose	Trial Length	Patients	Phases of Clinical Trials	Main Results	Reference
Omega-3 fatty acid docosahexaenoic acid as a triglyceride ester	Create a food product enriched with omega-3 fatty acids to examine its influence on fatty acid composition of blood cell membranes.	EPA: 570 mg/pouch DHA: 1900 mg/pouch.	28 days	10	Phase II	Consumption of the food product was well-tolerated by participants and resulted in a statistically significant decrease in C-reactive protein levels.	[22]
Omega-3 fish oil	Investigate the relative efficacy of HU and folic acid (standard therapy) compared to the addition of omega-3 or vitamin D supplementation in managing the frequency and severity of painful crises in individuals with sickle cell disease.	Capsules of either 1000 mg omega-3 fish oil (400 mg EPA and 300 mg DHA) or 1.5 mL vitamin D (2800 IU/7 mL)	12 months	165	Phase III	The omega-3 group exhibited a significant differential change in serum lipid profile compared to the control group, characterized by an increase in high-density lipoprotein (HDL) and low-density lipoprotein (LDL).	[23]
Vitamin A	Investigate the potential benefits of high-dose vitamin A supplementation in mitigating clinical complications associated with SCD in children.	3000 or 6000 IU/d	8 weeks	42	Pilot Study	Neither 3000 nor 6000 IU/d of vitamin A were sufficient to increase serum vitamin A concentrations in children with SCD.	[24]
EvenFlo and folic acid	Evaluate the potential benefits of a combined nutraceutical supplement (EvenFlo + folic acid) compared to standard folic acid supplementation alone in managing the clinical manifestations of SCD.	Folic Acid 500 microgram (mcg) OD and EvenFlo 500 milligram (mg) BID.	4 weeks	61	Phase I	EvenFlo in addition to folic acid is an effective agent in the management of SCD.	[25]
Vitamin D	Evaluate the potential benefits of a combined nutraceutical supplement (EvenFlo + folic acid) compared to standard folic acid supplementation alone in managing the clinical manifestations of sickle cell disease (SCD).	Daily 1000 IU vitamin D3	10 months	369	Phase III	In children with SCD, combining a high-dose vitamin D bolus with daily 1000 IU vitamin D3 supplementation demonstrated greater efficacy in elevating 25-hydroxyvitamin D levels compared to daily supplementation alone.	[26]
Arginine	Evaluate the efficacy and safety of oral L-arginine (Arg) therapy in reducing the severity of vaso-occlusive painful crises in children with sickle cell anemia within the Nigerian population.	100 mg/kg/dose	24 moths	68	Phase II	Children receiving oral L-arginine therapy experienced a significant reduction in both the duration of vaso-occlusive crisis resolution and the length of their hospital stay.	[27]
Arginine	Investigate the impact of L-arginine supplementation on Doppler-derived indices of cardiopulmonary hemodynamics in children with sickle cell anemia experiencing vaso-occlusive pain crisis.	300 mg/kg/d in three divided doses (100 mg/kg/dose)	22 months	66	Phase II	L-arginine supplementation demonstrably improves cardiopulmonary hemodynamics in children with SCD experiencing vaso-occlusive pain crisis and acute chest syndrome.	[28]
Voxelotor	Investigate the differential pharmacokinetic and pharmacodynamic profiles of voxelotor in healthy adults compared to patients with SCD.	Doses of 100, 400, 1000, 2000, or 2800 mg.	72 days	48	Phase I/II	Voxelotor acts by increasing hemoglobin’s affinity for oxygen, promoting the stabilization of the non-sickling, high-oxygen affinity (oxy) conformation of hemoglobin S, thereby reducing its propensity to polymerize and form sickle-shaped red blood cells.	[29]
Voxelotor	Conduct a comparative evaluation of voxelotor’s efficacy and safety against placebo in adolescents and adults with SCD, assessing its impact on hemoglobin levels, hemolysis markers, and the frequency and severity of vaso-occlusive crises.	1500 mg and 900 mg	72 weeks	274	Phase III	Voxelotor treatment resulted in a statistically significant increase in hemoglobin levels and a concurrent reduction in markers associated with hemolysis in patients with SCD.	[30]
Hydroxyurea	Investigate the efficacy of hydroxyurea as a primary stroke prevention strategy in children with SCD within the Nigerian population.	Low-dose (10 mg/kg per day) or moderate-dose (20 mg/kg per day)	20 months	220	Phase III	A low to moderate dose of hydroxyurea reduces the frequency of vaso-occlusive episodes in the hospital and acute pain incidents at home.	[31]
Acetaminophen and Diclofenac Sodium	Evaluate the effectiveness of intravenous acetaminophen versus intravenous diclofenac sodium in treating skeletal VOCs in children with SCD.	Intravenous acetaminophen at 10 mg/kg/dose 8 hourly and intravenous diclofenac sodium at 1 mg/kg/dose 8 hourly	2 months	104	Phase I	Intravenous acetaminophen offers a more effective treatment option than intravenous diclofenac for managing skeletal VOCs in children.	[32]
Canakinumab	Examine the hypothesis that persistent inflammatory processes associated with SCA contribute to the spectrum of clinical manifestations experienced by patients in an ambulatory setting.	300 mg (or 4 mg/kg for participants < 40 kg) canakinumab (provided as 150 mg/1 mL of liquid in vials) or matching placebo	6 months	47	Phase II	This study proposes that canakinumab’s targeted inhibition of IL-1β-driven inflammation in adolescents and young adults with SCA might offer therapeutic advantages with minimal safety risks.	[33]
Ticagrelor	Evaluate the efficacy and safety of ticagrelor, a reversible P2Y12 inhibitor, compared to placebo in preventing VOCs in pediatric patients with SCD.	Three different doses of ticagrelor were given based on body weight: 12 to 24 kg: 15 mg (1 tablet) twice daily; 24 to 48 kg: 30 mg (2 tablets) twice daily; 48 kg: 45 mg (3 tablets) twice daily.	8 moths	193	Phase III	Ticagrelor treatment did not demonstrate a significant reduction in the rate of VOCs compared to placebo in pediatric patients with SCD.	[34]
Ketamine	Investigate the potential benefits and safety profile of single-dose ketamine infusion for managing acute VOCs in adult patients with SCD.	Ketamine (0.3 mg/kg) in 100 mL of normal saline.	12 months	278	Phase III	Single-dose ketamine administration in adult SCD patients with acute VOCs led to a significant decrease in pain scores within a 2-h timeframe.	[35]
Isoquercetin	Investigate the potential of isoquercetin in mitigating thromboinflammation associated with SCD.	28 to 35 doses of 1000 mg	32 months	46	Phase II	Potential to reduce a range of thromboinflammatory biomarkers within the context of SCD management.	[36]

**Table 2:** Potential therapeutic effects of bioactive compounds from natural products in sickle cell disease.

Herbal Products	Bioactive Compounds	Main Functions	References
Garlic	Organosulfur compounds (such as allicin, alliin, methylsulfanylalmethane, and diallyl disulfide). Flavonoids (such as quercetin, catechin, and epicatechin).	Targeting hemolysis-mediated endothelial dysfunction. Antioxidant potentials. Infective conditions especially respiratory infections in SCA. Significant enhancement in erythrocyte deformability by stabilizing the membranes of non-sickled red blood cells.	[5,98,99,100]
Black beans (*Phaseolus vulgaris* L.)	Anthocyanins, flavonols, flavones, and tannins.	Antioxidant properties. Inhibitory and reversibility activities on sickling. Stability effect on the membranes of erythrocytes. Targeting HbS polymerization.	[5,98,101]
*Carica papaya* seed oil	Phenolic compounds, fatty acids, tocopherols, and carotenoids.	Antioxidant activity. Effective in enhancing human sickle cell blood polymerization inhibition in females. Increase catalase activity in female human sickle cell blood.	[98,102,103,104]
Garcinia kola Heckel	Saponins, phenolic compounds, and flavonoids.	Targeting vaso-occlusion: modulate inflammatory responses. Effective membrane stabilization effects. Antisickling and antioxidant activities.	[5,71,98,105]
*Cajanus cajan* L. (seed)	Flavonoids and phenolic compounds.	Increase in β-globin synthesis and oxyhemoglobin concentration. Decrease in rate of polymerization and percentage hemolysis of the red blood cells. Antisickling effect.	[106,107,108,109]
*Zanthoxyllum heitzii* (Rutaceae)	Phenolic compounds (syringic acid, vanillic acid, protocatechuic acid, and p-hydroxybenzoic acid) and alkaloid compounds.	Antisickling property. Antioxidant properties. Antiradical activity. Sickle cell disease polymerization inhibition and sickle erythrocyte membrane stabilization.	[109,110,111]

## Data Availability

The data presented in this study are available on request from the corresponding authors.
